# Deep-Eware: spatio-temporal social event detection using a hybrid learning model

**DOI:** 10.1186/s40537-022-00636-w

**Published:** 2022-06-28

**Authors:** Imad Afyouni, Aamir Khan, Zaher Al Aghbari

**Affiliations:** University of Sharjah, Sharjah, United Arab Emirates

**Keywords:** Social Data Mining, Event Classification, NLP, Deep Learning, Spatio-Temporal Scope, Stream Data Management

## Abstract

Event detection from social media aims at extracting specific or generic unusual happenings, such as, family reunions, earthquakes, and disease outbreaks, among others. This paper introduces a new perspective for the hybrid extraction and clustering of social events from big social data streams. We rely on a hybrid learning model, where supervised deep learning is used for feature extraction and topic classification, whereas unsupervised spatial clustering is employed to determine the event whereabouts. We present *‘Deep-Eware’*, a scalable and efficient event-aware big data platform that integrates data stream and geospatial processing tools for the hybrid extraction and dissemination of spatio-temporal events. We introduce a pure incremental approach for event discovery, by developing unsupervised machine learning and NLP algorithms and by computing events’ lifetime and spatial spanning. The system integrates a semantic keyword generation tool using KeyBERT for dataset preparation. Event classification is performed using CNN and bidirectional LSTM, while hierarchical density-based spatial clustering was used for location-inference of events. We conduct experiments over Twitter datasets to measure the effectiveness and efficiency of our system. The results demonstrate that this hybrid approach for spatio-temporal event extraction has a major advantage for real-time spatio-temporal event detection and tracking from social media. This leads to the development of unparalleled smart city applications, such as event-enriched trip planning, epidemic disease evolution, and proactive emergency management services.

## Introduction

Event Detection from Social media has been studied intensively over the last decade, with the aim of understanding people’s interests, feedback, check-ins, and social happenings with regard the hot topics discussed on daily bases over social networks [2, 9]. The content generated by users is massive and rich; therefore, researchers, stakeholders, and authorities can build applications to extract insightful spatio-temporal information about live events of interest (EoI).

A ‘social event’ can be commonly defined as the occurrence within a specified space and time of a real-world unusual happening [18]. For example, when a social attraction occurs (e.g. a new festival or a start of a shopping discount season) in the city of Dubai, a large number of tweets will be posted about such a social event. Social events usually comprise family reunions, promotions, incidents, announcements, or natural hazards, among others. The dynamic updates of such events by the live communities in social media lay the ground for developing plenty of intelligent location-based services (LBSs). These LBSs can support a variety of domain applications, such as trip planning, emergency management, transportation, navigation, city exploration, education, and crime intelligence [23, 24].

Existing studies on event detection aim at detecting specific or generic types of events, but generally focus on extracting the main topic or subtopics related to global events [4]. However, extracting other features that describe the evolution and spanning of such events over space and time need further investigation. In addition, existing techniques can find events from snapshots of historical social data in offline mode, but fail to consider the big data aspect and stream processing of such events. Existing event detection systems do not fully support big data stream processing, which is mandatory to achieve a scalable and worldwide event extraction and visualization.

This paper presents a different perspective for the event discovery from social networks using a hybrid learning approach, that aims not only at discovering the surrounding happening using a deep learning model, but also infer the spatio-temporal belonging and tracking of clusters discussing such dynamic events. We propose *‘Deep-Eware’*, a big data system based on hybrid learning model for the extraction and tacking of socially-enabled spatio-temporal events. We built a convolutional neural network (CNN) based deep learning (DL) classifier, along with a bidirectional LSTM model to train and classify the collected tweets. Later, an unsupervised learning model is developed, where events are clustered by identifying anomalous topics that are spatially-related and within a given time period. The spatio-temporal clustering performs grouping of similar or semantically-linked tweets by considering the textual features and the temporal patterns of the sentences. Detected topic clusters will undergo a hierarchical spatial *‘de-clustering’* in order to obtain the final event clusters that are tagged with spatial and temporal components.

The *Deep-Eware* platform comprises a scalable architecture that lays the ground for efficient mining of big social data streams, by leveraging cutting-edge big data and stream management tools (e.g. Spark, Kafka, Apache Nifi, GeoServer, etc.). *Deep-Eware* provides a seamless integration and visualization of clustered events on a worldwide map, thus allowing for a unique city exploration enriched with live spatio-temporal events.

Our platform lays the ground for the development of unparalleled smart city applications including smart trip planning, tracking and prediction of major events, such as epidemic disease evolution (e.g., COVID-19 as an example), and proactive emergency management services. When compared to related literature, the main contributions of this work are summarized as follows:

1. Developing a hybrid learning mechanism to extract social events and to monitor their spatio-temporal extents.
2. Designing an automated technique to generate training datasets for event classification using deep learning, by enriching the semantics of extracted data using KeyBERT, rather than only employing word frequency or occurrence-based string matching.
3. Implementing a fully-fledged big data system, referred to as *‘E-ware’*, that integrates data management and processing tools for the spatio-temporal event discovery.
4. Evaluating our E-ware platform with respect to efficiency and effectiveness of results. We assess the intrinsic properties of our algorithms for the detection, scalability, and clustering accuracy, among others.

The remainder of this paper is organized as follows. “Related work” section discusses the related work on event extraction from social media in the bid data era, while “System overview” section provides an overview of the system and describes its salient components. Our data preprocessing and ingestion methodology is discussed in “Event classification methodology” section. “Spatio-temporal clustering and location inference” section introduces the details behind the spatio-temporal event detection technique, and the spatio-temporal data management within the framework is discussed in “Data Stream ingestion” section. “Implementation details” section highlights some implementation details, and then, “Evaluation” section presents the evaluation and discussion on results. Finally, concluding remarks are highlighted, showing the potential of this research.

## Related work

Event detection from social media can provide deeper insights about user’s and community interactions on a variety of unspecified topics of interest [7, 32]. This section presents the main related work on event detection and social data processing techniques.

### Detection of social event of interest

Social events of interests, which will be referred to simply as events in the rest of the paper, can be observed as the representation of the real-world happenings at a given location and time. These happenings can be classified based on the *thematic* (e.g., festival or sport events), *temporal*, *spatial*, and other learning features (e.g., user profiles and social links) [2, 27, 29]. Discovering and disseminating events from diverse online social networks and with a variety of modes (e.g., text, image) have been the focus in many research studies, such as politics [1], traffic analysis [5], and fashion analysis [26]. Existing works on event detection aim at detecting specific [5, 12] or generic types of events [22]. However, extracting other features that describe the evolution and spanning of such events over space and time need further investigation [35]. In addition, existing techniques can find events from snapshots of the social data streams ignoring the incremental and continuous development of such events [34].

Approaches and models for event extraction from social media mainly include feature-pivot (based on temporal features of data), document-pivot (i.e., classify documents on based a given similarity measures, such as, TF-IDF or Cosine similarity), and topic modeling (e.g., Latent Dirichlet Allocation) [4, 19]. Event extraction usually comprises three stages: (1) data filtering and preprocessing; (2) data representation that involves evaluating the significance of words in incoming streams or data batches; and (3) clustering phase. The authors in [20] introduced a model for event detection without manual labeling in the training data, by injecting a bias in the Neural Network with an Attention Mechanism. A comprehensive survey summarizing all recent related work in this topic can be found in [2].

Ahuja et al. [3] proposed a model for spatio-temporal event detection (STED) by employing a probabilistic approach to discover events by their associated topic, occurring time, and spatial occurrence from news and Twitter data sources. Although this latest work present several advantages and has a similar objective to our study, its focus was on detecting and monitoring the global events that were discussed on news, rather than a generic model to discover all types of events. Also, there was no discussion on how to maintain a continuous processing of data streams in order to update event clusters. Another recent work on spatio-temporal event detection has introduced the principle of incremental processing over temporal slices (i.e., hours, days, and weeks), and spatial resolutions (i.e., cities, regions, and countries) [25]. However, this work only considered and monitored specific event domains (e.g., elections, sports), rather than a general purpose event detection technique. A Power-law distribution model applied to spatio-temporal data was presented in [16]. Two algorithms were introduced, where a basic version could only represent time-series data at multiple spatial resolutions, while an advanced version could apply semantic similarity over tweet content to generate more meaningful events. George et al. [15] used quad-tree structure for hierarchical partitioning of space, and the Poisson distribution model to detect streams’ density.

The proposed technique presents a unique approach that takes advantage of the latest technologies in data stream management, to discover spatio-temporal events. Our system automatically updates the spatial and temporal scopes of extracted event clusters with upcoming data streams, which are divided based on temporal slices. The spatial distribution of events is also calculated in a hierarchical manner, in order to understand the significance of such events based on users’ interaction.

### Deep learning for event detection

Lately, deep learning methods are used to detect social events. The work in [11], maps tweets into numerical feature vectors using word-embedding models and then supervised deep-learning algorithms including convolutional neural network (CNN) and recurrent neural network (RNN) are used to traffic events. The work in [21] proposed a Pairwise Popularity Graph Convolutional Network (PP-GCN) model to classify social events. The model takes two weighted inputs: meta-path instance similarity and textual semantic of social posts. Graph Neural Networks has been utilized by [8] to detect social events incrementally. They proposed a Knowledge-Preserving GNN model used social graphs to group social posts and facilitate incremental processing. In [33], the authors used Deep Belief Network (DBN) and Long Short-Term Memory (LSTM) to extract traffic-related frequent keywords from social posts to build association rules. These rules are then used to detect traffic accident events. Collective social events are detected to understand social, political, and economic processes of people, however they are challenging to detect. Bidirectional Encoder Representations from Transformers (Bert) is used build the DeepLuenza model to accurately identify influenza reporting tweets by the work in [6]. DeepLuenza may provide early insights about influenza outbreaks. The work in [31] utlized Convolutional neural networks (CNNs) to analyze image data and recurrent neural networks (RNNs) with long short-term memory to analyze text data of social posts in a two-stage classifier model to detect social collective action events.

### Performance and scalability perspectives

Implementing large-scale event detection requires digesting large volumes of data streams, and should consider big data management and near-real-time processing techniques. Traditional data analysis algorithms and techniques do not scale in high computational complexity with large datasets in social media. The rise of parallel and distributed computing, mainly leveraged by the MapReduce paradigm [10], has enabled an unprecedented use of big data mining tools and machine learning techniques in a variety of domains. For instance, Apache Hadoop and Spark, are tangible implementations of the MapReduce paradigm. Other distributed file systems that are commonly using the MapReduce paradigm are Apache Pig, Apache HDFS, and Stratosphere. Besides, data stream processing is leveraged by several open-source tools including Apache Kafka, Apache Storm, Spark Streaming, and Flink. Libraries for machine learning are Apache Mahout, SparkMLlib, and MLBase.

Data stream management allows for a continuous manipulation and processing of unbounded streams coming from real-time sources. Several spatial and spatio-temporal indexing schemes have been proposed in non-relational distributed databases [28, 30]. A real-time trend detection and monitoring model from social media was recently presented in [14]. However, their focus was on extracting trending topics rather than individual spatio-temporal events. Fuzzy clustering with an adaptive classification of tweets using Apache Spark was also presented in [17].

## System overview

**Fig. 1:**
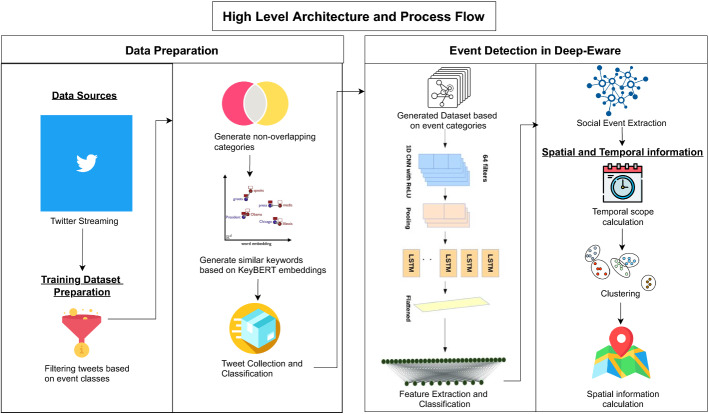
System architecture

Our system presents a fully integrated solution to detect events in the region. It handles unstructured data from social media (Twitter) and plots the events on an interactive map. The methodology of the system is presented in Fig. 1. A brief overview of each component is presented below.

- Building our dataset: Collecting tweets of each category with a unique approach to avoid manual labeling.
- Preprocessing the data: encode tweet texts into embeddings vectors using Bert.
- Training the algorithm: training a deep learning algorithm to classify tweets into different categories.
- Events clustering: cluster incoming events using Spatio-temporal clustering. Supervised clustering of known classes and Unsupervised clustering of unknown classes.
- Map visualization: locate events on the map.

## Event classification methodology

To be able to classify tweets into events, we built a classifier model, which involved the phases as shown in Fig. 1.

### Building the dataset

#### Identify event classes

Creating knowledge bases for *events* is expensive, time-consuming, and subjective. The existing knowledge bases are domain dependent and may have limited coverage. None of the existing knowledge bases is related to *events*. Therefore, we decided to extract the event classes relying on a wide coverage online encyclopedia developed by a large number of users, namely Wikipedia. By taking the Wikipedia categories as input, we extracted all event-related categories and their subcategories in the Wikipedia category structure. As a result, a list of main events is created.

#### Generate non-overlapping categories

It is common in knowledge bases, including Wikipedia, to have categories that may partially cover the same topic. Those are called overlapping categories. In some applications, these overlapping categories may compliment each other, and not considered to be duplicating topics. For event detection purposes that we consider in this paper, we plan to only extract non-overlapping categories. From these extracted categories, we created a list of main events. The list contained 12 different types of events, which are: COVID-19/Health, Politics, Economy, Education, Incidents, Jobs, Promotions, Religion, Sports, Tourism, Weather, and Celebrations.

#### Keyword generation

After generating the main categories of events, we augmented the list by finding all synonyms and semantically similar words to the extracted categories. That is we utilized the advantage of the KeyBERT,^1^ which is based on SBERT embeddings to generate keywords and key phrases from a document based on the most similar tokens relative to that document. For each main category’s word, a set of similar keywords are generated. For example, collecting keywords of the word sport will extract a list of keywords from the document related to the sports.

#### Tweet collection

The training data was collected from Twitter. We used python’s Twitter API (tweepy) to collect tweets using different searching queries. We collected 25,530 tweets for training purposes. In addition, we were targeting tweets with more than 4 words. The selection of 4 words as a minimum of a tweet is for the purpose of collecting meaningful sentences.

Moreover, We collected a test dataset using the streaming Twitter API. The test dataset contained 4703 geotagged tweets. Table 1 shows the number of tweets for each category. We tried to collect balanced groups of tweets to ensure the model will not be biased during training. As compared to previous works, the collected tweets in this work, have more categories, and each category consists of more tweets.

**Table 1:** Number of Tweets for events and non-events categories

		Category	Number of Tweets
Events	1	COVID-19/Health	2000
	2	Politics	2000
	3	Economy	2000
	4	Education	2000
	5	Incidents	1918
	6	Jobs	2000
	7	Promotions	2000
	8	Religion	2000
	9	Sports	2000
	10	Tourism	2000
	11	Weather	2000
	12	Celebrations	1793
Non-Events	Others		1819
	Total		25,530

### Tweet pre-processing

After collecting the tweet dataset, the tweets are preprocessed to remove irrelevant content for classification. In particular, the tweets undergo the following steps:

- Non-English character: each tweet is processed to remove all non-English characters from the text of the tweet. Non-English characters include URLs, emojies, etc.
- Repeated characters: some users repeat character to emphasize a certain concept, or meaning. However, such repeated character are irrelevant to event classification. For examples, the letter *x* is repeated in the word *exxxxxellent*.
- Stop words: the existence of stop word in English, like in other languages, might negatively effect the classification of tweets in their correct event classes. Therefore, we remove all stop words from the tweets. Examples of stop words are: *the, in, for, etc.*
- Common words: we have eliminated the most commonly repeated words in our dataset. Some words like, name of countries *UAE, France, Egypt, UK, etc.*

The algorithm for determining the event class, and event properties, and for converting unstructured streams into potential candidates with stream heading and content works as follows (see Algorithm 1).
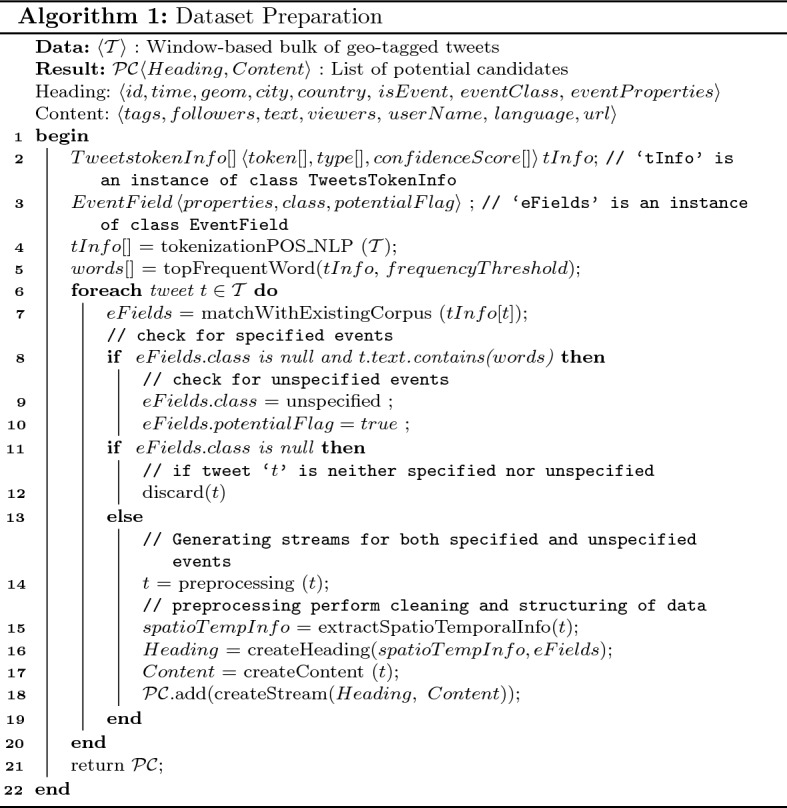


During data integration and preprocessing, the quality of stream content is also checked based on the following parameters:

- *Exactness of data*: data from multiple sources comes with different levels of correctness. Data from authority has a higher priority and trust level as compared to other sources. Incorrect data can be hazardous, so we need to check the exactness of data in order to make sure that no streams are generated by software bots, for instance.
- *Duplication removal*: Many chunks of data can be retrieved several times, as in the case of a weather streaming source, that continuously publish new streams on the current weather status for a given city. So, we need to remove duplication in case of multiple streams related to the same event and from the same source.
- *Data dependency*: Some information provided by one source may be incomplete and requires more data to extract relevant knowledge [13]. For example: from tweets, we get information about an accident but after analyzing data from physical sensors, we can get details of the severity and impact of that accident.

### Tweet representation

**Fig. 2:**
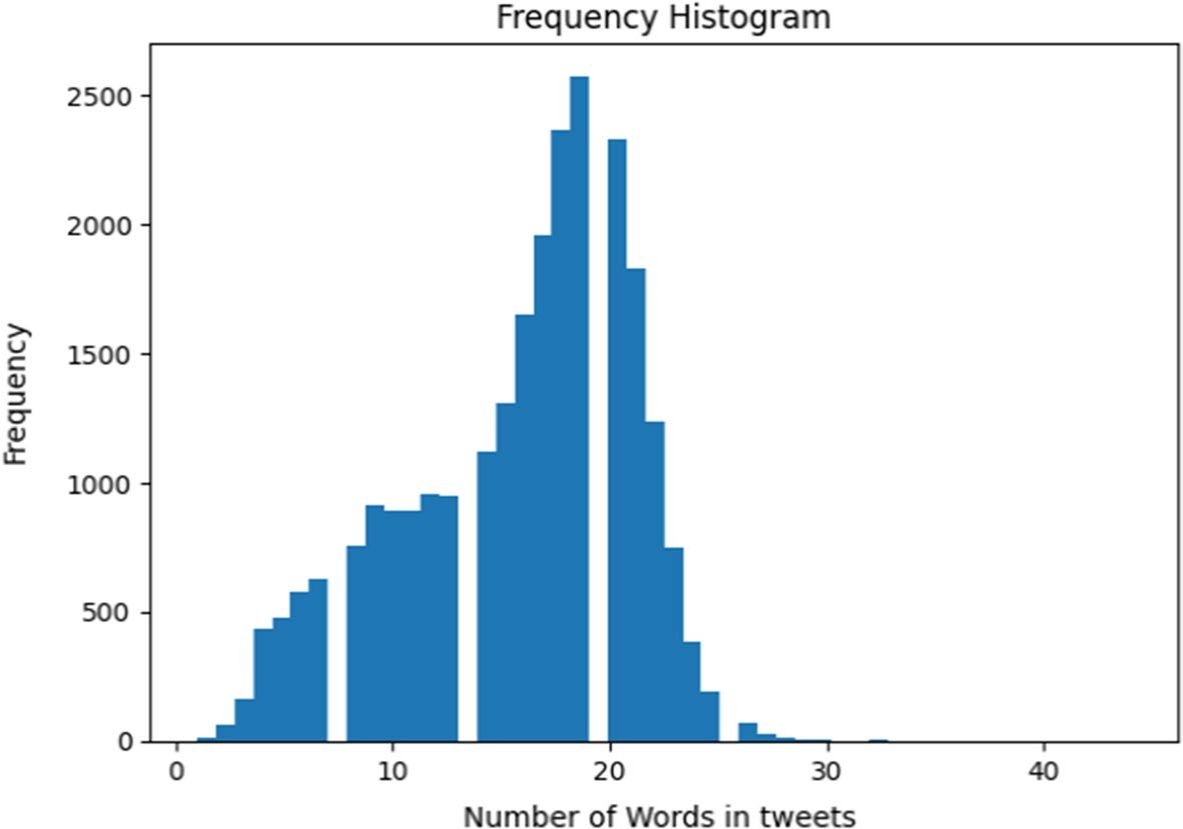
Frequency of number of words in tweets

**Fig. 3:**
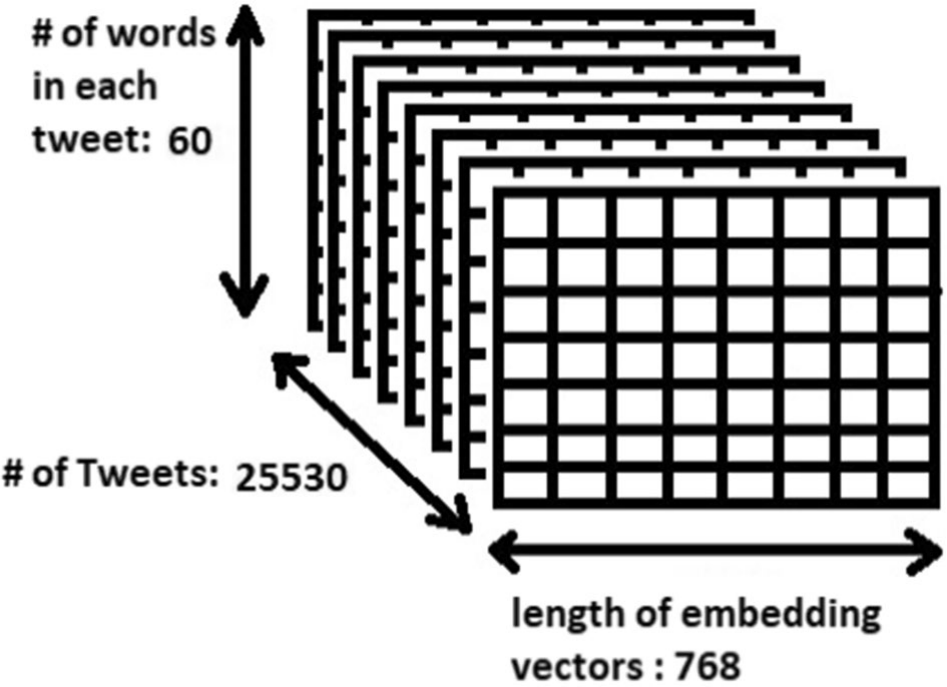
3D arrays of preprocessed tweets before feeding to the algorithm

After preprocessing, each resulting tweet would contain a set of English words, called *tokens*. Each of these tokens is processed by BERT embedding to convert it into a vector of numbers. Therefore, a tweet is represented by a set of vectors equal to the number of words in the tweet. Each of these vectors is of length 768. Also, we used padding to these vectors to make them of equal length. As shown in Fig. 2, each tweet in the current collected dataset has a maximum length of 30 words. We decided to use 60 as padding length to handle any tweets in the future. It requires 113.3 mins to preprocess the 25,530 tweets on our machine. Thus, the dataset of tweets if of size (25,530, 60, 768) before feeding the tweets to the Deep Learning classifier, Where 25,530 is the number of tweets, 60 is the number of words in each tweet, and 768 is the length of the embedding vector. Figure 3 shows a 3D presentation of the preprocessed tweets.

### Training the Algorithm

#### Tweet labeling based on event categories

The training dataset is categorized and labeled based on the above mentioned categories, and manual verification was performed over all tweets to make sure they are well labeled and no overlapping between categorized tweets has occurred. The *‘others’* set of tweets was built by making sure they do not belong to any of the 12 specified classes. For the testing dataset, we randomly collect a set of tweets with no prior keywords or drivers, so that classification is performed on a completely random set of Twitter streams.

#### Training with the generated dataset

**Fig. 4:**
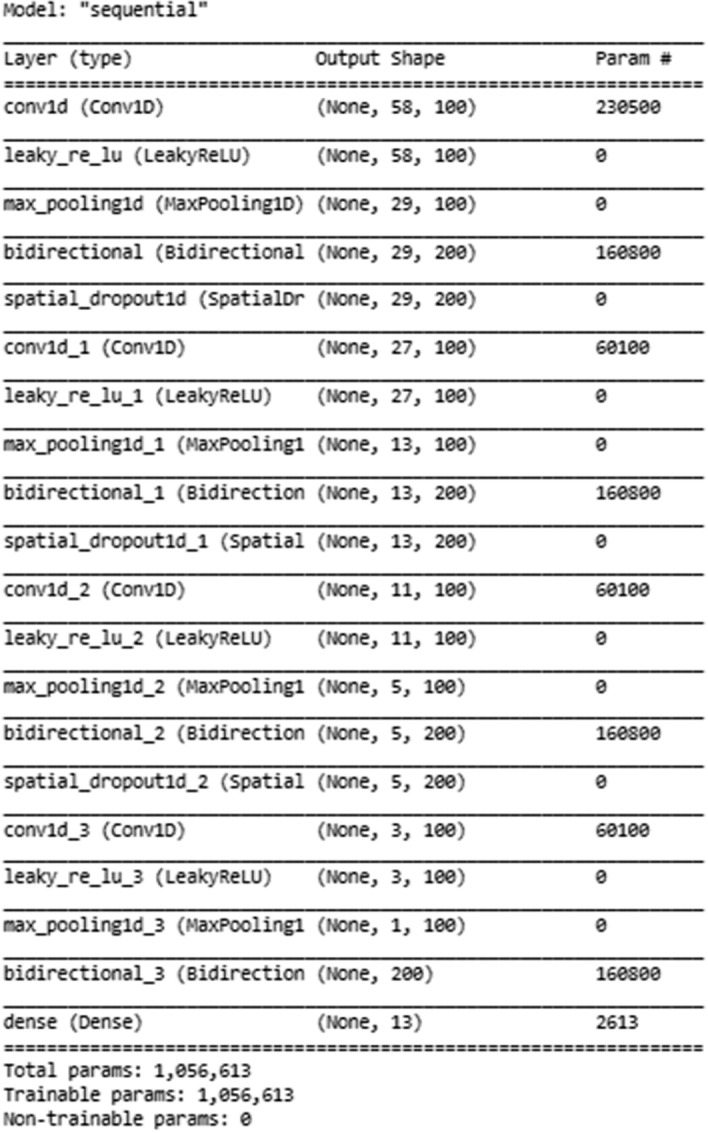
Deep learning structure

We built a convolutional neural network (CNN) based deep learning (DL) classifier model to train and test the collected tweets. Figure 4 shows the DL structure of the model. The CNN architecture consists of several layers, such as Conv1d, LeakyRelu, Maxpooling, Bidirectional LSTM, and Spatial Dropout layers. The model consists of four groups of these layers followed by a dense layer of 13 Neurons and SoftMax activation function. Moreover, the model uses the Adam Optimizer algorithm, and the Categorical Cross-Entropy Loss function. Furthermore, the model uses 0.00001 L2 regularization in Conv1d layers and a kernel size of 3. The alpha value is 0.2 in leakyRelu layers and 2 as pool size in Maxpooling 1D layers.

We were using a GPU in google Colab as a training and testing environment. The training time was 13.33 min. The dataset of tweets was split into 70% for training and 30% for validation.

#### Deep learning model validation

To validate the CCN model shown in Fig. 3, we computed the validation accuracy on 30% of the validation subset of tweets. Figure 3 shows the training/Validation loss and accuracy. The model had reached more than 80% accuracy in the training and validation accuracies. While training and validation, losses reached 0.75 with 20 epochs.

**Fig. 5:**
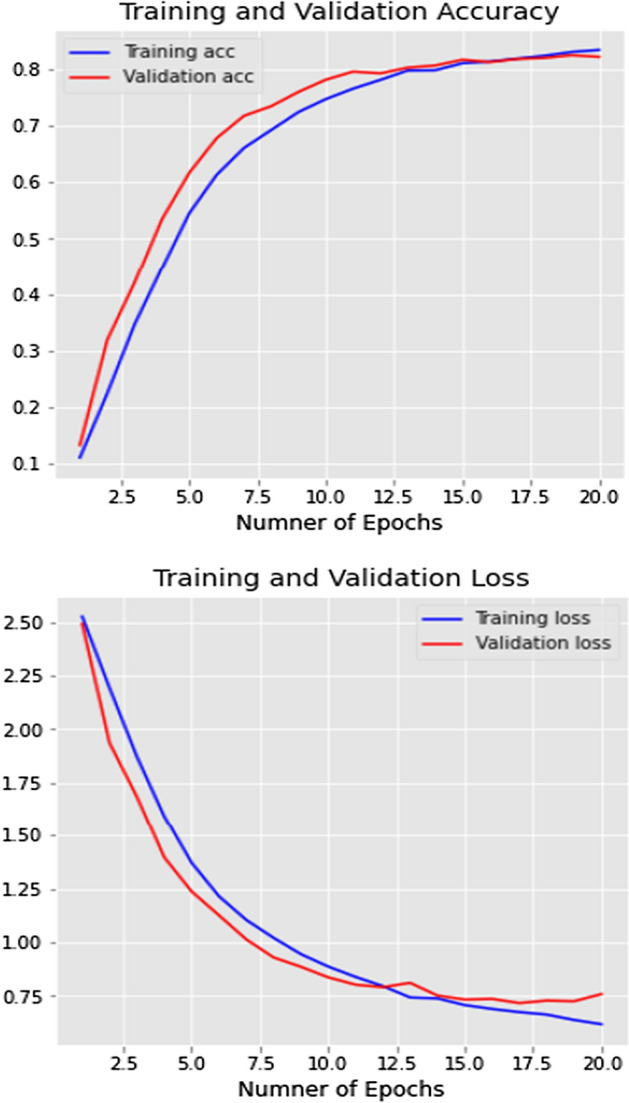
Training loss and accuracy

## Spatio-temporal clustering and location inference

### Spatio-temporal clustering

To detect events that evolve over space and time, the proposed model performs spatio-temporal clustering on the incoming tweets. As a result, a cluster of tweets, which are within close spatial distance and within relatively short time, is considered an event. We used the st-dbscan library in python to perform spatio-temporal clustering. We used parameter values of 0.1, 1000, and 5 for epsilon1, epsilion2, and minimum sample size, respectively. The results were validated using the the proposed deep learning model, which confirms that it can be used to classify unseen tweets with high accuracy.

#### Mapping spatio-temporal clusters to pre-difined categories

After applying Spatio-temporal clustering, we passed the tweets of one Spatio-temporal cluster to the proposed deep learning model to classify them into pre-defined categories. Then, tweets of similar topics, or categries, are combined if they close in terms of space and time. So, each event will have the same Spatio-temporal cluster and the same topic.

#### Identifying new classes of spatio-temporal clusters

For tweets that are not clustered into the pre-defined categories, they undergo spatio-temporal clustering and then unsupervised topic creation. First, we tried a naive approach to identify the topic of the tweets of each spatio-temporal cluster. In this approach, we collect similar tweets by finding pairs of tweets whose similarities are above a certain threshold. This process continues hierarchically until we reach *K* number of clusters. But, it was too complicated and has a combinatorial complexity. For example, if we have a list of three tweets [1,2,3]. To form a group of similar tweets, the similarity of tweet 1 should be computed to tweet 2 and 3, and the similarity of tweet 2 should be computed to the similarity of tweet 3. Imagine if we have 5 tweets and we want to get all possible groups of similar tweets. The algorithm should perform all similarity computations between all pairs to find similar group of tweets. Therefore, we decided to use topic modeling with BERT, that we perform topic clustering and topic identification. It takes as input, a preprocessed tweet (see Sect. 3) and encode the tweets into 768 embedding vectors. Then, cluster these vectors that represent the tweets with HDBSCAN. Clusters with at least 5 tweets are considered as valuable clusters, and ignored clusters with less than 5 tweets. Finally, topics are created from the considered clusters by extracting the most frequent terms in the cluster. These frequent terms of a cluster forms the topic of the cluster. In our experiments, we took the top 15 frequent words to represent the cluster.

**Fig. 6:**
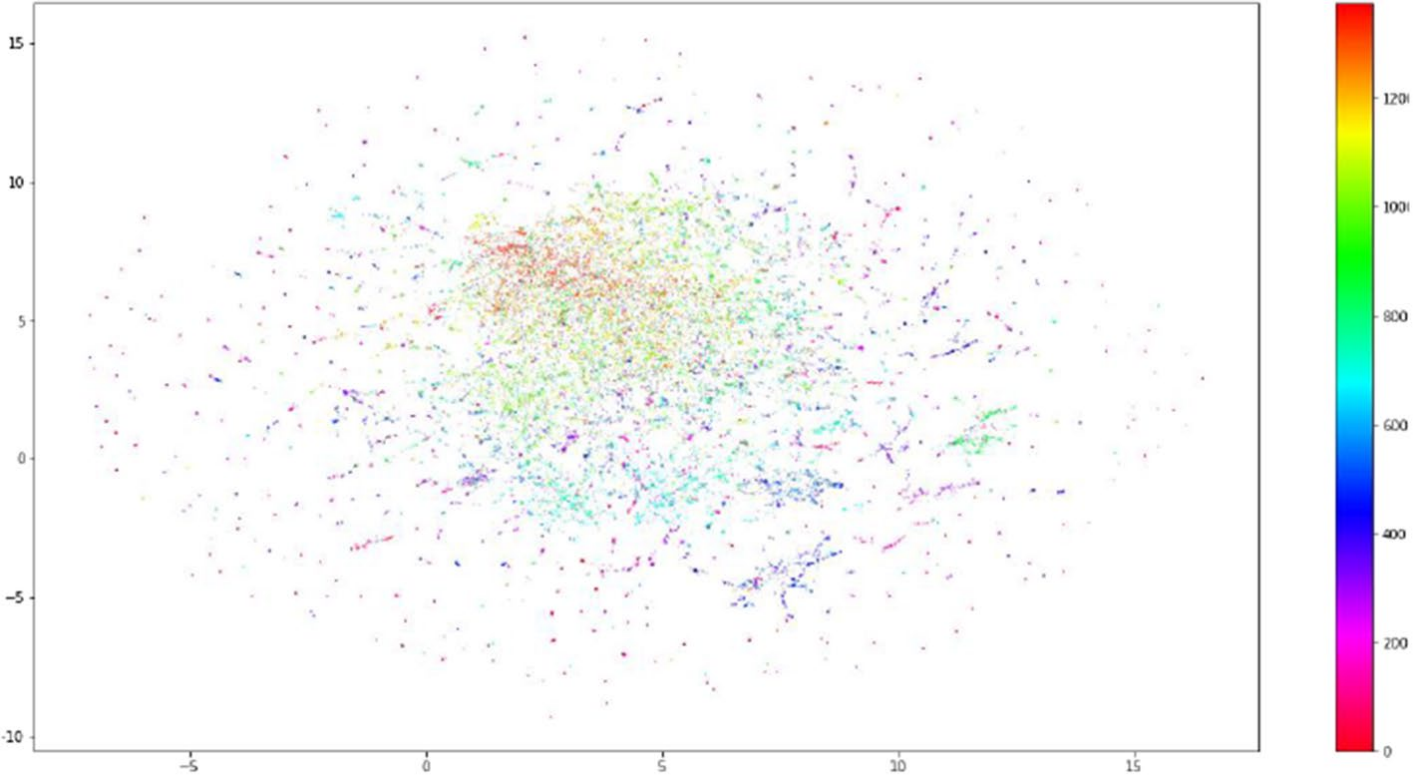
Topic distribution of the training dataset

Figure 6, shows an example of topic distribution and visualization of tweets. It shows clusters of the training dataset. We can see around 12 main classes appear with different colors.

### Location inference

To infer locations of tweets, we implemented two methods. First method depends on dividing the target area into zones, and then the method identifies the source zone of a tweet. This method is relatively accurate but time-consuming. While the second method, depends on extracting a location from the tweet’s metadata. This method is less accurate but gives faster results.

#### Method 1: Divide Target Area into Zones

**Fig. 7:**
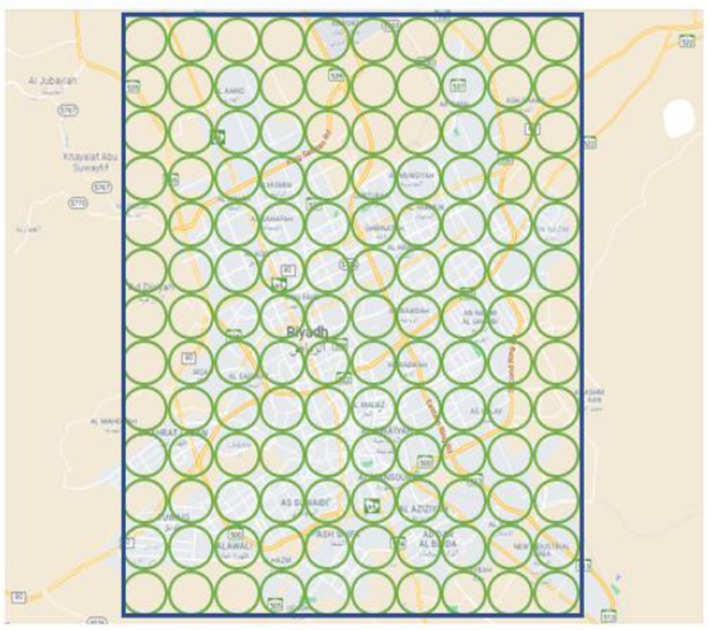
Location inference by dividing the area into small circular zones

The first method divides the target area into small circles, called zones. Then, the method identifies the source zone of a tweet. The center of the source zone of a tweet is considered the location of the tweet. This method is relatively more accurate but consumes more time. Figure 7, shows an example of this approach, where the city of Riyadh, KSA, is divided into small circular zones of radius 100 m. The input tweets were retrieved from the city of Riyadh and mapped to those zones.

#### Method 2: Extract Locations from Tweet’s Metadata

The second method extracts a location of a tweet from the tweet’s metadata using Tweepy API. The location of a tweet is represented as boundary boxes of the source area. Therefore, we consider the center of this box to be the location of the tweet. Although this method is less accurate than method 1, but it more practical and can easily be automated to extract the locations of the input tweets. Therefore, in the proposed model, we used method 2 to infer the locations of the input tweets.

## Data Stream ingestion

The data streams are collected from Twitter. Different platforms such as Instagram and Flickr can be incorporated by preparing similar Kafka topics as input, and by performing multi-source data fusion at the beginning of the pipeline or after the event detection process. The Data Preprocessing and Ingestion phase involves three major steps: Filtering, Packaging and Ingestion.

For ingestion, we use Apache Kafka to build a fault-tolerant Big Data pipeline. The Kafka producer reads streams from twitter and publish them to Kafka topics. The data from Kafka is consumed in real-time. The tweet properties are packaged into a Kafka payload. Kafka payload is published to a Kafka topic. Additional components for data crawling and filtering can be added for new data sources without modifying other components, due to the fact that Kafka allows multiple topics to be consumed separately and concurrently. Hence, a different data filter should be written for each new source which is added to the system. This allows our system to digest different types of data input, and to generate structured data forms out of unstructured streams.

### Spatial de-clustering of aggregated topics

The spatial extent depicts the whereabouts of the extracted event. Since all the tweets are geo-tagged, their point coordinates help us in estimating the event location. We consider topic-related tweets as the initial input for the final geo-event discovery. We integrate a spatial clustering technique applied to the coordinates of all tweets to cluster those related to the same topic in a close proximity. We have used HDBSCAN clustering algorithm since it supports the haversine distance that computes distances on a sphere between geo-locations. Also, with HDBSCAN, there is no need to indicate the number of resulting clusters in advance, as it processes data points hierarchically. Two main parameters are used to characterize how HDBSCAN should perform the hierarchical clustering: *r* for radius, which specifies the spatial range in meters and helps in merging clusters within a specified distance, and the $min-cluster-size$ that specifies the minimum size allowed for a group of data points to form a cluster. These parameters are manipulated and tested in order to assess the performance of our event detection approach as a whole, and in particular, the clustering accuracy of final events.

We refer to this process as *de-clustering* because it is classifying tweets that belong to one topic based on the spatial dimension. Since we have only considered the geo-tagged tweets (latitude and longitude), each tweet will have a spatial attribute associated with it. Every topic has related tweet(s). If related tweets count is more than 1 then spatial clustering can help with the identification of events from these topics.

To form an event, we group the tweets by *class* and *spatial cluster id*. Each of these groups is assigned an id called the *event id*. In the above example: three different tweets at three different locations that belong to the topic cluster ‘view, opening, hiring, read, latest, job, sales’, will be formed as three different events with different event ids.

From the above process, we get spatio-temporal clusters that are tagged by class. An event is formed for each of these clusters, and is assigned an event id. The final event clusters are determined with a set of related topics, temporal evolution and spatial clustering. These extracted events are published to another topic in Kafka in a continual manner for the pipeline to work smoothly.

**Fig. 8:**
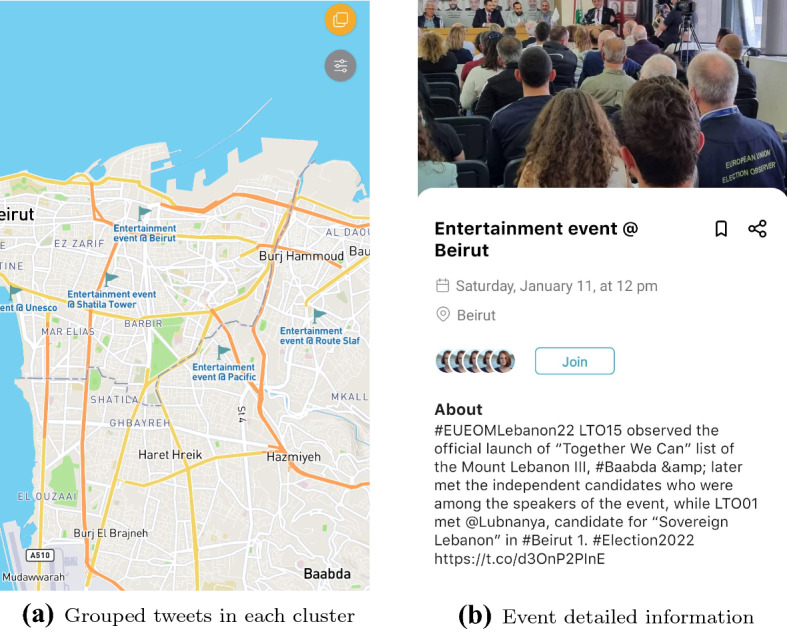
Details from extracted events

After the events cluster integration, we add the newly detected events to Geomesa-Accumulo database. The Apache Kafka and Apache NiFi tools are used for the ingestion pipeline. Since we already use a ‘topic’ to store streaming tweets in Apache Kafka, we built other ‘event’ topics to store newly detected events. The NiFi process is built to automate the dataflow between the components. The term ‘dataflow’ here depicts the automated and managed flow of information from Kafka into Geomesa–Accumulo database. The Geomesa-Accumulo database schema is defined before any data is transferred from Kafka to Geomesa through NiFi.

**Fig. 9:**
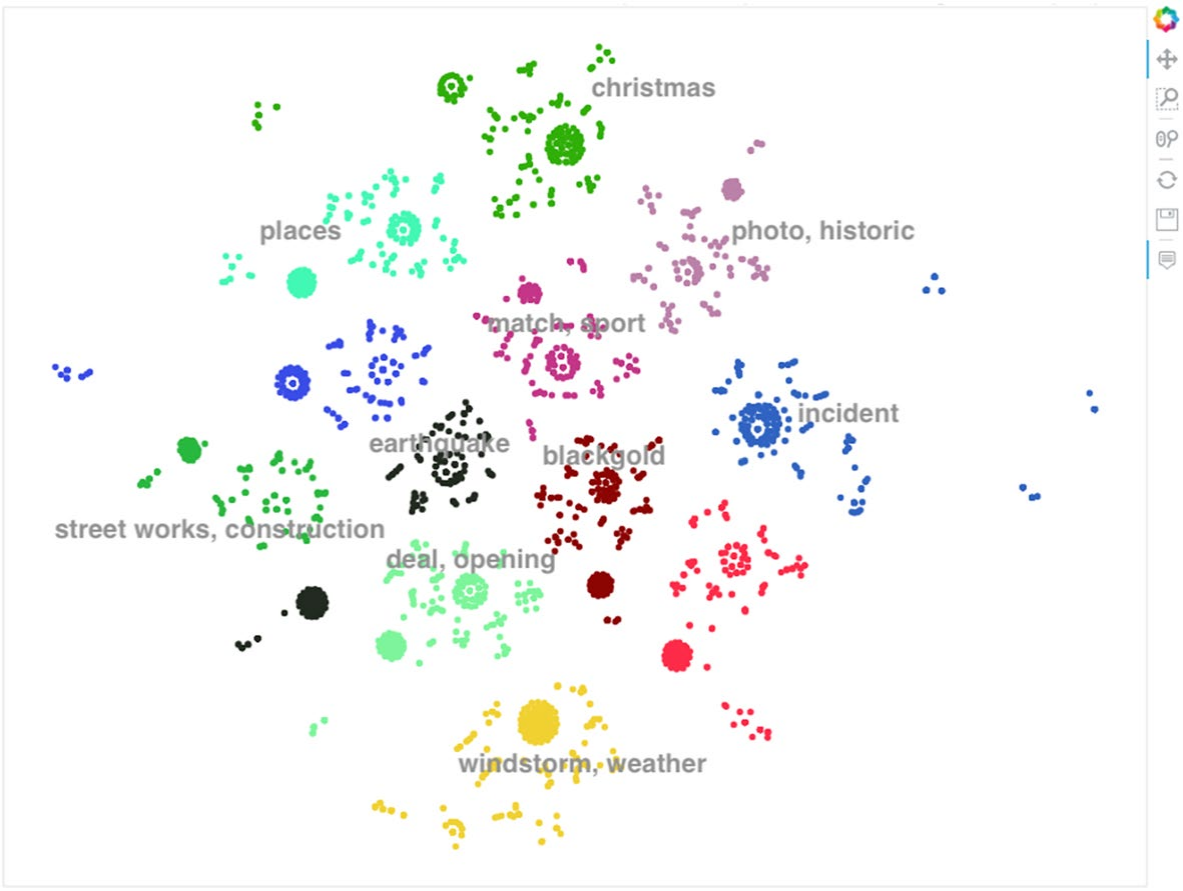
Cluster visualization of Deep-Eware trained on 8137 tweets, 12 unspecified topics, and thresholding at 0.7 topic probability

## Implementation details

In this section, we discuss the implementation of Deep-Eware as a big data Processing system, and the event visualization module. Figure 8a and b demonstrates the event extraction visualization on maps, with detailed description of the event. Moreover, topic-based visualizations can be demonstrated in Deep-Eware, where all events are categorized within major topics as illustrated in Fig. 9. In this figure, multiple events from different locations, and at different period can share the same topic, so they are grouped together only based on textual features.

### Deep-Eware Big Data processing

The proposed system was setup on Ubuntu 18.04 operating system in a standalone mode. The device used was a MSI GS40-6QE laptop with the following key specifications: Intel Core i7-6700HQ processor, 16GB DDR4-2133 RAM, and 1TB Samsung 840 Evo Solid-State Drive. We used the following versions of the tools to setup the proposed system: Java version 8, Python version 3.6, Zookeeper version 3.4.10, Apache Kafka version 2.11-2.1.1, Apache NiFi version 1.8.0, Hadoop version 2.8.4, Apache Accumulo version 1.9.2, Geomesa-Accumulo verison 2.3.1, and Geoserver version 2.14.4. It is worth mentioning that all the required tools are open source.

This dataset consists of tweets with geolocations collected using the Twitter Streaming API. We use Twitter’s streaming API to collect data in real-time. Tweets are streamed from Twitter Streaming API and are fed into Apache Kafka. The data from Kafka is consumed in real-time. Python was used to write the Kafka Consumer. Python’s tweepy library was used to stream the data from Twitter.

For NiFi to upload the events data to Geomesa–Accumulo store, we connected two processors, Kafka and Geomesa–Accumulo in the NiFi’s user interface. We defined the required specifications for NiFi to understand the data from Kafka, and required converters to convert the data from Kafka for Geomesa to understand it. This was the ingestion pipeline.

## Evaluation

This research contains two parts, the first part is tweet content classification using a deep learning approach (supervised learning). Then, the second part is events extraction using Spatio-temporal clustering. According to Fig. 5, we have reached more than 80% accuracy on training and validating data.

We have tested the model on a new streamed batch from Twitter (4703 tweets) to evaluate the model. Since the system will get streamed tweets and will be used to plot events on an interactive map. We have made a threshed of 70% to acquire a single label. The number of tweets of a single label is 4400, while 303 tweets had either two labels or multi-labels. For example, some tweets could contain political speech and economical speech at the same time. Some of them contain political and religious speech. While multi-label categories contain more than 2 categories.

Table 2 shows the evaluation measures (Recall, Accuracy, Precision and F1 are represented in Eqs. 1–4) of each category of the supervised learning approach. The ‘Actual’ column contains the number of tweets of each category in the testing dataset. We can see the accuracy value is high since the number of tweets in each category is low compared to ‘others’ class. In general, people are using words not related to the same category to describe their topic, which affected the recall, precision, and F1. For example, people were using political words to describe sports events. Then these tweets came under the political category while they are sports tweets and so on. Then we used an unsupervised approach to classify the streamed tweets. Table 3 shows the evaluation results of the unsupervised learning approach. We got 47 clusters that represent a single label out of 163 clusters. Some of these clusters are talking about sport, politics, promotions, etc. We have an additional category where clusters have the same tweets talking about an event that happens (Special Events clusters). For example, some of these clusters are talking about the Chinese rocket which fell to the earth, or the World Migratory Bird Day, and some competitions related to this day. Then we have calculated the accuracy of each cluster and got the average of accuracies to evaluate the category. We can see some drop in the average accuracy as we have some weak clusters affecting the average accuracy of the category. For example, in politics category, we have 5 clusters with high accuracies (80%, 88%, 100%, 100%, 100%) and 5 clusters with medium accuracies (71%, 71%, 73%, 75%, 78%) and 3 clusters with low accuracies (44%, 50%, 57%). So, the average will be 76% and so on with other categories.

1 $$\begin{aligned}&Accuracy = \frac{TP+TN}{TP+FP+FN+TN} \end{aligned}$$

2 $$\begin{aligned}&Precision = \frac{TP}{TP+FP} \end{aligned}$$

3 $$\begin{aligned}&Recall = \frac{TP}{TP+FN} \end{aligned}$$

4 $$\begin{aligned}&F1 Score = \frac{2*(Recall* Precision)}{(Recall + Precision)} \end{aligned}$$

**Table 2:** Evaluation results of the supervised learning approach

Category/evaluation measure	Actual	Recall	Accuracy	Precision	F1
COVID-19 /Health	18	1.00	1.00	0.70	0.82
Politics	146	0.78	0.99	0.97	0.86
Tourism	41	0.53	0.99	0.98	0.69
Sports	69	0.82	0.99	0.86	0.84
Economy	44	0.88	1.00	0.98	0.92
Jobs	25	0.68	1.00	1.00	0.81
Promotions	10	0.26	0.99	1.00	0.41
Incidents	123	0.95	1.00	0.99	0.97
Religion	74	0.84	1.00	0.99	0.91
Celebrations	17	0.76	1.00	0.94	0.84
Education	29	0.87	1.00	0.90	0.88
Weather	3	0.17	1.00	0.67	0.27
Others	3801	1.00	0.98	0.95	0.98
# Bi-labels / Multilabels Tweets				303	
Total Tweets				4703

**Table 3:** Evaluation results of the unsupervised learning approach

Category	# Clusters	# Tweets in clusters	# Correct tweets in clusters	Average accuracy
Politics	13	177	128	0.76
Economy	4	28	21	0.79
Sports	5	52	36	0.58
Religion	14	125	111	0.91
Education	1	14	8	0.57
Promotions	2	14	10	0.75
Special Events	3	27	18	0.70
COVID-19 /Health	2	25	16	0.64
Tourism	3	21	13	0.62
Others	116	4220		
Total	163	4703		

### Effect of events’ spatial clustering radius

To measure the effect of modifying the radius of event clusters on the performance of event detection, we computed the Precision, Recall and F1 of the resulting event clusters. For this experiment, we fixed the minimum cluster size to 5 and the number of time slices to 10. The manual annotations of clusters was performed independently by three human subjects.

**Fig. 10:**
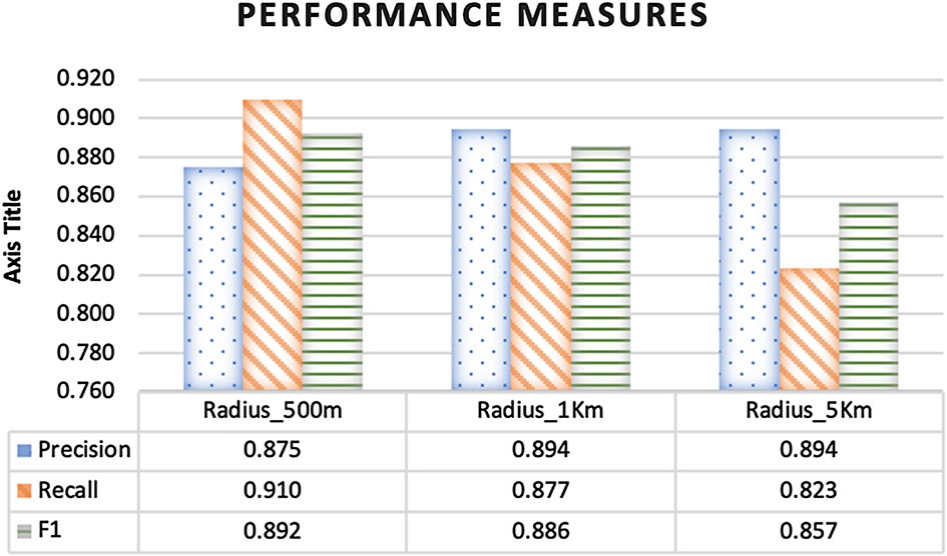
Effect of varying the event clustering radius on the performance measures: Precision, Recall and F1

Figure 10 shows the computed Precision, Recall and F1 at three different radii, 0.5 km, 1 km and 5 km. Note that Precision and F1 performed best at 1 km. This is expected since as the radius becomes too large, cluster grow large, which in turn increases the possibility of more irrelevant events being included in the cluster. Furthermore, when the radius becomes too small the clusters are caused to be fragmented. In these fragments, the false positives become more dominant, which affect the homogeneity of the clusters and thus lowers the Precision.

On the other hand, Recall decreases as the radius becomes larger, i.e. clusters become larger. When clusters become larger, small clusters may be encapsulated in big ones, and this may result in an increased number of false negatives. Therefore, the clustering recall is negatively affected. Nonetheless, as clusters become larger they tend to include more relevant events, which improves the precision measure as shown in Fig. 10.

**Fig. 11:**
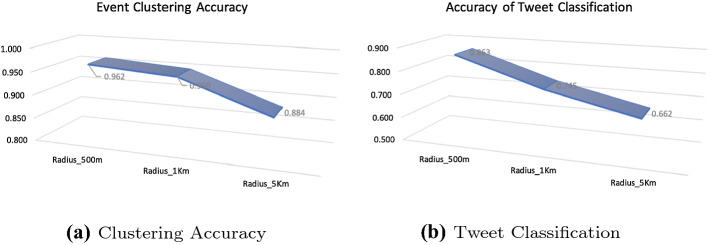
Effect of varying the radius in spatial clustering

In Fig. 11 we show the effect of modifying the radius of events’ spatial clusters on the Accuracy of clustering and tweet classification. As the radius increases, the clusters become larger and thus they become more likely to contain irrelevant events (false positives). Therefore, the accuracy of clustering drops as shown in Fig. 11a. Similarly, the accuracy of tweet classification describes how many raw tweets were actually correctly clustered within the event clusters. The degradation of performance shown in Fig. 11b is due to the fact that a large number of independent tweets may be falsely classified as part of an event because of the big radius specified.

The results above demonstrates that our platform can achieve good performance in terms of incremental event detection accuracy, clustering quality, and also in terms of efficiency to compute in near-real time over continuous sliding windows of data streams.

## Conclusion

This paper investigated the hybrid learning approach for discovering spatio-temporal events from social media. The system is based on a scalable and efficient big data platform that can manage and mine a data flow of unstructured streams by relying on the state-of-the-art big data and stream management tools (Spark, Kafka, Apache Nifi, geoServer, etc.). We introduced Deep-Eware, a scalable architecture for social data mining based on deep learning and spatio-temporal clustering. Dataset generation was automated with word embedding and keyword generation tools. Other approaches only consider one snapshot of historical data, and miss the temporal or spatial component when extracting events. Deep learning and NLP algorithms were developed in this research on top of transformer-based word embeddings (KeyBERT) in order to leverage unbiased feature extraction for the event detection process. Results of the event detection performance and clustering technique show important improvement in terms of mapping those events spatio-temporally to be visually located on digital maps. Diverse types of applications can be implemented on top of Deep-Eware, such as, event-enriched trip planning, and tracking and forecasting of natural disasters and epidemics, among others.

## Footnotes

1 https://github.com/MaartenGr/KeyBERT.
